# Supplementary material for: The genome sequence of the Lesser noctule, Nyctalus leisleri (Kuhl, 1817) (Chiroptera: Vespertilionidae)
Source: Wellcome Open Res. 2026 May 30;11:323. [Version 1] doi: 10.12688/wellcomeopenres.26704.1 (PMC13560838; doi:10.12688/wellcomeopenres.26704.1)
Supplement: Peer review history

# Reviewer report: Reviewer response for version 1

**DOI:** 10.21956/wellcomeopenres.29405.r161173

**Published:** 2026-08-13

**Authors:** Jiang Feng

**Affiliations:** 1 Northeast Normal University, Changchun, China

This work is a standardized genome data note produced as part of the Darwin Tree of Life and Bat1K consortia. The authors successfully assembled a high-quality, haplotype-resolved chromosome-level reference genome for the lesser noctule (Nyctalus leisleri), which provides valuable foundational resources for future research on vespertilionid evolution, migratory adaptation, and conservation genomics of threatened bat species. Several minor textual and descriptive revisions are still required to further improve the overall rigour and readability of this manuscript.

 1.I recommend inserting a concise subsection at the end of the Methods chapter to clarify that gene prediction for this species was carried out using the standard Ensembl mammalian annotation pipeline. Briefly outline the core steps of this pipeline, including repeat masking, integration of multi-omics transcript evidence, and gene model prediction. A formal reference to the official Ensembl annotation methodology should also be included to enhance the reproducibility of this study.

 2.Two conflicting descriptors for the specimen appear across the text: the term “juvenile” is used in one section, while “adult” is adopted elsewhere. Please revise the manuscript to unify this terminology and eliminate contradictory descriptions of the sample’s developmental stage.

 3.At present, Figure 3 only labels chromosome IDs along the axes without explanatory text in its legend. Two key pieces of information should be added to the figure caption. 1)Context explaining the original sorting rule for chromosome numbering, to resolve the numerical inconsistency between chromosome length and naming order mentioned in the main text. 2)Explicitly mark the positions of the X and Y sex chromosome pseudomolecules within the Hi-C contact map, enabling readers to rapidly identify sex-chromosome scaffolds.

 4.The lengthy paragraph introducing Table 4 in the Results only lists the source and benchmark origin of the metrics, rather than presenting core research outcomes. Please move all this descriptive background text into the legend beneath Table 4. Retain only one concise concluding sentence in the main Results text.

Are sufficient details of methods and materials provided to allow replication by others?

Yes

Is the rationale for creating the dataset(s) clearly described?

Yes

Are the datasets clearly presented in a useable and accessible format?

Yes

Are the protocols appropriate and is the work technically sound?

Yes

Reviewer Expertise:

animal ecology, conservation biology

I confirm that I have read this submission and believe that I have an appropriate level of expertise to confirm that it is of an acceptable scientific standard, however I have significant reservations, as outlined above.

# Reviewer report: Reviewer response for version 1

**DOI:** 10.21956/wellcomeopenres.29405.r161168

**Published:** 2026-07-29

**Authors:** Xiuguang Mao

**Affiliations:** 1 East China Normal University, Shanghai, China

This manuscript presents a high-quality chromosome-scale genome assembly for *Nyctalus leisleri*, which will be valuable for comparative genomics in Vespertilionidae as well as for the conservation of this species. For the current version of the manuscript, I have two comments:

 1) The authors state that "these chromosome-level scaffolds, confirmed by Hi-C data, are named according to size (Figure 3; Table 3)." However, in Table 3, Chr3 (212.18 Mb) is longer than Chr2 (206.95 Mb), which contradicts the stated naming criterion.

 2) The manuscript concludes with a genome annotation report for this species; however, no detailed annotation procedures were provided prior to this report.

Are sufficient details of methods and materials provided to allow replication by others?

Yes

Is the rationale for creating the dataset(s) clearly described?

Yes

Are the datasets clearly presented in a useable and accessible format?

Yes

Are the protocols appropriate and is the work technically sound?

Yes

Reviewer Expertise:

Comparative genomics, genome assembly, evolutionary biology, speciation, bats

I confirm that I have read this submission and believe that I have an appropriate level of expertise to confirm that it is of an acceptable scientific standard.

# Reviewer report: Reviewer response for version 1

**DOI:** 10.21956/wellcomeopenres.29405.r158993

**Published:** 2026-07-08

**Authors:** Edgar G Gutierrez

**Affiliations:** 1 National Polytechnic Institute, México, Santo Tomás, Mexico

Overall, this manuscript makes a valuable contribution by presenting a high-quality genome assembly and annotation that will serve as an important resource for future comparative and evolutionary genomic studies. The manuscript is well written, the analyses are appropriate, and the results are presented clearly. The genome assembly is of high quality, and the accompanying analyses effectively demonstrate its value as a genomic resource. Most of my comments are intended to improve the clarity, organization, and consistency of the manuscript. In particular, I recommend reducing unnecessary repetition and providing additional methodological details where appropriate (e.g., the genome annotation workflow and key software parameters). Addressing these points will improve the readability and reproducibility of the study without affecting its main objectives.

**Abstract**

             The transition between the description of the mitochondrial genome and the nuclear gene annotation is ambiguous. In the sentence beginning with "Gene annotation of this assembly...", the phrase "this assembly" appears to refer to the mitochondrial genome described in the preceding paragraph rather than the nuclear genome. Please rephrase this sentence to clarify that the reported 18,985 protein-coding genes belong to the nuclear genome assembly.

**Introduction**

- Please rephrase the sentence describing the species as both "widely distributed" and "generally rare," as these expressions may appear contradictory without additional context.
- In the section discussing threats, consider merging this sentence with the preceding paragraph to improve the flow of the text.
- In the final paragraph, consider shortening the description by removing details that are not directly relevant to the origin and preservation of the specimen.
- In the sentence beginning "The specimen was collected and identified by Gail Armstrong (Bat 1 K)...", the acronym "Bat1K" appears to contain spaces between the characters. Please verify whether this is a formatting issue.

**Methods**

- In the "Nucleic acid extraction" section, consider shortening the procedural description by omitting details that are not essential for reproducibility and placing greater emphasis on the DNA quality metrics relevant to sequencing.
- Please use a consistent level of detail when describing instruments throughout the Methods section, including manufacturer and model where appropriate. For example, the Qubit Fluorometer and the FemtoPulse system are currently described with different levels of detail.
- Similarly, please ensure consistency in the description of instruments, kits, and quality-control procedures across the DNA, Hi-C, and RNA library preparation sections.
- For reproducibility, briefly describe the metrics assessed by Gfastats, BUSCO, and MerquryFK when these tools are first introduced.
- The description of Pretext Snapshot is missing a citation. At minimum, please include a reference or hyperlink to the GitHub repository, as has been done for several other software tools.
- In the BlobToolKit workflow, consider citing all software used in the analysis to maintain a consistent and complete methodological description.
- Please specify the BUSCO lineage dataset (e.g., *laurasiatheria_odb10*) in the Methods section rather than introducing it only in the Results. In addition, consider removing the BUSCO version number from the Results section, since it is already provided in Table 5. This would improve consistency by presenting methodological details in the Methods while reserving the Results.
- I suggest adding a brief subsection describing the genome annotation workflow, or at least citing the Ensembl annotation pipeline. Although the annotation was performed by Ensembl, referencing the underlying methodology would improve transparency and provide readers with the necessary context to interpret the annotation results.
- Consider removing software version numbers from the Methods section, as both the versions and URLs of all programs are already provided in Table 5. This would reduce redundancy and improve the overall readability of the manuscript.

**Results**

- Figure 2 shows the proportion of unique sequence but does not explicitly report the estimated repeat content. Consider indicating that the repeat content was derived from the GenomeScope model, or report the estimated value directly in the figure to improve clarity.
- The sentence introducing Table 4 describes the table contents and data source rather than presenting a study result. Consider moving this information to the table legend and using the Results section to emphasize the reported EBP assembly metrics and their significance.

Are sufficient details of methods and materials provided to allow replication by others?

Partly

Is the rationale for creating the dataset(s) clearly described?

Yes

Are the datasets clearly presented in a useable and accessible format?

Yes

Are the protocols appropriate and is the work technically sound?

Yes

Reviewer Expertise:

Genomics, immunogenetics, bioinformatics.

I confirm that I have read this submission and believe that I have an appropriate level of expertise to confirm that it is of an acceptable scientific standard, however I have significant reservations, as outlined above.
